# Global incidence of lip, oral cavity, and pharyngeal cancers: Disparities, determinants, and opportunities for prevention, early detection, and treatment

**DOI:** 10.3322/caac.70059

**Published:** 2025-12-23

**Authors:** Fernando Neves Hugo

**Affiliations:** ^1^ Department of Epidemiology and Health Promotion New York University College of Dentistry New York New York USA

Studying cancer incidence by country presents a unique global health opportunity to investigate lip, oral, and pharyngeal cancers (LOPCs), aiming to identify international disparities and understand disease burden across various levels of socioeconomic development. This cross‐national approach highlights the central role that social determinants play in shaping LOPC burden inequalities worldwide.^1^ LOPCs constitute a heterogeneous group of diseases with distinct etiologic profiles and anatomic subsites. Oral cavity cancers are primarily linked to tobacco use in its various forms and alcohol consumption, whereas oropharyngeal cancers share these traditional risk factors but are increasingly associated with high‐risk human papillomavirus (HPV) infection, particularly HPV types 16 and 18.^2^

The findings reported by Rumgay and colleagues^3^ in this issue of *CA: A Cancer Journal for Clinicians* provide a comprehensive overview of LOPC incidence across 185 countries, using estimates from the International Agency for Research on Cancer. The authors estimated 758,000 new cases of LOPC globally in 2022 with a predominance of men (7:1 ratio), with cancers of the oral cavity, followed by the oropharynx and nasopharynx, representing the most incident cases. Their results reveal that oral cavity cancer incidence remains alarmingly high in South‐Central Asia, Melanesia, Micronesia, and Polynesia; whereas oropharyngeal cancer rates are elevated in Europe, and represent the most common LOPC subsite among men in North and South America.^3^

Given the distinct etiologies of oral and oropharyngeal cancers, these findings call for equitable health promotion and prevention strategies tailored to specific risk exposures within each country. Rumgay and colleagues^3^ appropriately emphasize the World Health Organization (WHO) Framework Convention on Tobacco Control and its MPOWER measures (Monitoring tobacco use; Protecting people from tobacco smoke; Offering help to quit tobacco use; Warning about the dangers of tobacco; Enforcing bans on tobacco advertising, promotion, and sponsorship; and Raising tobacco taxes) as central tools to reduce exposure. Emerging evidence links comprehensive implementation of MPOWER policies with significant reductions in global smoking prevalence and consequent declines in incidence, mortality, and disability‐adjusted life‐years related to LOPCs.^4^, ^5^

Efforts to curb alcohol consumption and increase adoption of gender‐neutral HPV vaccination are both feasible and essential but have shown uneven progress compared with efforts to reduce consumption of tobacco products. In this regard, the WHO Global Alcohol Action Plan 2022–2030 and the SAFER initiative provide evidence‐based, cost‐effective strategies. However, evaluations indicate challenges, such as commercial interference, limited political will, and resource constraints—especially in low‐income and middle‐income countries—that hinder full implementation. These obstacles particularly affect LOPCs, in which alcohol remains a modifiable risk factor because its reduction could substantially decrease future cancer burden.

Regarding HPV vaccination, a recent systematic review indicated that 148 WHO member states have incorporated HPV vaccines into their national immunization programs, achieving population‐weighted, average first‐dose coverage of 61.6% and full‐dose coverage of 47.6% among girls aged 9–14 years. Despite progress, global HPV vaccination coverage remains below the WHO target of 90% by 2030, with considerable variation by region and income. Notably, initiatives like the Gavi Vaccine Alliance have expanded access significantly, protecting over 86 million girls in lower income countries by 2025. Coverage for boys lags substantially, especially where immunization programs prioritize girls only.

The importance of LOPC screening should not be neglected. It represents an opportunity for early identification, with important effects in terms of better treatment outcomes and overall greater survival. Despite its potential, current challenges include inconsistent implementation, limited evidence of definitive mortality benefits, and variable accuracy of screening techniques. Visual examination is the most commonly used method. Although evidence from large studies has demonstrated promising results in terms of reducing oral cancer mortality among high‐risk individuals,^6^ evidence supporting the adoption of screening strategies for oropharyngeal cancer remains extremely limited. Furthermore, evidence to support the use of adjunctive technologies, including toluidine blue and brush biopsy to reduce LOPC mortality, is currently lacking.^7^

In relation to screening for LOPCs, the gaps are clear. There is a need for high‐quality, large‐scale studies to establish the true effect of screening on mortality, alongside standardized protocols that enhance diagnostic accuracy. Future efforts should focus on refining screening techniques, expanding training programs for health care providers to support the incorporation of screening into workflows, and developing evidence‐based guidelines that reconcile resource limitations with the urgent need for early detection. Integrating oral cancer screening into primary health care and oral health care, supported by ongoing research and technological innovation, represents a unique opportunity to reduce the global burden of LOPCs. In addition, comprehensive health technology assessments that integrate clinical effectiveness, cost‐effectiveness, ethical, social, and organizational dimensions are essential to systematically compare and identify optimal health promotion, prevention, and screening strategies to reduce LOPC burden while ensuring equitable access and maximizing health system sustainability.

The results reported by Rumgay and colleagues^3^ underscore the necessity of context‐specific, multifaceted strategies to reduce LOPC incidence worldwide by addressing tobacco‐related, alcohol‐related, and HPV‐related risks through comprehensive and equitable public health interventions. The success of countries that implemented MPOWER provides a roadmap and reinforces that the successful implementation of public policy has the potential to reduce exposure to important risk factors, with potential benefits in terms reducing the LOPC burden. The development of novel, affordable screening tools to support early diagnosis of LOPCs, particularly oropharyngeal cancer, along with an increase in the uptake of HPV vaccination and the adoption of upstream approaches to reduce exposure to well established risk factors for LOPCs, has the potential to importantly shift the trend of LOPC and reduce existing between‐country disparities.

## CONFLICT OF INTEREST STATEMENT

The author declares no conflicts of interest.
